# Modeling Focused-Ultrasound Response for Non-Invasive Treatment Using Machine Learning

**DOI:** 10.3390/bioengineering8060074

**Published:** 2021-06-01

**Authors:** Tariq Mohammad Arif, Zhiming Ji, Md Adilur Rahim, Bharath Babu Nunna

**Affiliations:** 1 Department of Mechanical Engineering, Weber State University, Ogden, UT 84408, USA; 2 Department of Mechanical and Industrial Engineering, New Jersey Institute of Technology, Newark, NJ 07102, USA; zhiming.ji@njit.edu; 3 Department of Civil & Environmental Engineering, Louisiana State University, Baton Rouge, LA 70803, USA; mrahim6@lsu.edu; 4 Division of Engineering in Medicine, Department of Medicine, Brigham and Women’s Hospital, Harvard Medical School, Harvard University, Cambridge, MA 02139, USA; bnunna@bwh.harvard.edu or; 5 Advanced Energy Systems and Microdevices Laboratory, Department of Mechanical and Industrial Engineering, New Jersey Institute of Technology, Newark, NJ 07102, USA

**Keywords:** machine learning, numerical model, random forest, focused ultrasound, Rayleigh–Sommerfeld, angular spectrum

## Abstract

The interactions between body tissues and a focused ultrasound beam can be evaluated using various numerical models. Among these, the Rayleigh–Sommerfeld and angular spectrum methods are considered to be the most effective in terms of accuracy. However, they are computationally expensive, which is one of the underlying issues of most computational models. Typically, evaluations using these models require a significant amount of time (hours to days) if realistic scenarios such as tissue inhomogeneity or non-linearity are considered. This study aims to address this issue by developing a rapid estimation model for ultrasound therapy using a machine learning algorithm. Several machine learning models were trained on a very-large dataset (19,227 simulations), and the performance of these models were evaluated with metrics such as Root Mean Squared Error (RMSE), R-squared (R^2^), Akaike Information Criterion (AIC), and Bayesian Information Criterion (BIC). The resulted random forest provides superior accuracy with an R^2^ value of 0.997, an RMSE of 0.0123, an AIC of −82.56, and a BIC of −81.65 on an external test dataset. The results indicate the efficacy of the random forest-based model for the focused ultrasound response, and practical adoption of this approach will improve the therapeutic planning process by minimizing simulation time.

## 1. Introduction

Focused ultrasound is a reliable modality in many non-invasive therapeutic applications. It can be used to treat malignant tumors or cancerous cells in the brain, liver, kidney, pancreas, breast, prostate, and bone. In this technology, the ultrasound beam is focused inside a small volume of target tissue without affecting surrounding tissues or layers of tissues that are on the path of the beam [1]. The intense energy at the focused zone causes thermal coagulation and tissue ablation as the temperature increases [2]. Different non-invasive therapy uses different ranges of sonication times and pressure field intensities to achieve the desired outcomes [3,4]. Temperatures above 60 °C are used for typical surgical applications, and relatively lower temperatures (41 to 45 °C) are used for a more extended period of time in hyperthermia applications [5]. In addition, temperatures higher than 100 °C at the focus zone should be avoided, because superheated tissue can cause bubbles and/or explosive localized boiling at this temperature [6,7]. For these reasons, it is very critical to study and predict the effect of the pressure field and temperature rise at the focused zone during non-invasive ultrasonic treatment planning [8,9].

There are various commercial and non-commercial software available to predict ultrasound pressure field responses for homogeneous and heterogeneous media. The SPFlex module of PZFlex (Weidlinger Associates Inc., Mountain View, CA, USA) can create tissue maps from MRI images and then perform focused nonlinear wave simulation by finite element and explicit time-domain approach [10]. Other high-end finite element products like ANSYS and COMSOL can simulate focused ultrasound propagation, but they require significant user effort and computation power. In addition, the finite element approach does not produce a good solution and is significantly slower in the near-field region compared to numerical methods because of high-density mesh requirements in the near-field [11]. For these reasons, numerical methods such as Rayleigh–Sommerfeld integral and angular spectrum method are widely used to simulate focused ultrasound in tissue media. There are many other numerical methods used in this area, such as the Fast Nearfield Method (FNM) developed by McGough et al. [12,13,14] for circular, rectangular, and spherical pistons and hybrid angular spectrum developed by Vyas et al. [15].

The Rayleigh–Sommerfeld method, which is a popular approximation of Kirchhoff’s integral formula for the Helmholtz equation [16], produces relatively less error and can be considered as a reference for scalar wave propagations of ultrasound beam [17]. Therefore, the Rayleigh–Sommerfeld approach is a widely accepted method for focused ultrasound response calculations. However, it takes almost hours in a moderate speed workstation (Core i7, Dual-core 2.00 GHz processor, NVIDIA GeForce GT750M, and 16.0 GB RAM, Dell Inspiron i7737T, CA, USA) to simulate a time-harmonic beam within a homogeneous tissue volume of 100 × 50 × 20 mm^3^. To improve the calculation speed (about 20 min), another method known as angular spectrum can be used, where an already calculated 2D pressure field plane can propagate in a forward direction with the help of 2D Fourier transform. However, in both cases (Rayleigh–Sommerfeld and angular spectrum) the calculation time significantly increases (hours to days) if tissue inhomogeneity and layered or complex-shaped obstacles are included for more realistic simulations.

Existing numerical simulation results on a tissue model can be very useful for various reasons. Although every patient is different, their internal organs have similar arrangements and layers of tissues inside the body [18,19,20,21]. Thus, previous time-intensive simulations contain a huge amount of useful information that can be explored to reduce the treatment planning time.

The aim of this study is to develop an ultrasound response model to rapidly predict maximum pressure, maximum power deposition, and maximum temperature at the focused zone using machine learning algorithms. To achieve this goal, three machine learning algorithms (1) decision tree, (2) support vector regression, and (3) random forest were tested using Root Mean Squared Error (RMSE), R-Squared (R^2^), Akaike Information Criterion (AIC), and Bayesian Information Criteria (BIC) as performance metrics. In addition to machine learning models, a general linear regression model is used for comparisons. To train the machine-learning models, a large simulation dataset is created using a modified angular spectrum method. This modified angular spectrum method is developed in this study to achieve good results in numerical simulation for layered tissue media. In a previous study, we used a similar approach to find pressure fields in nonhomogeneous media using Rayleigh–Sommerfeld integrals [22]. Although the numerical computational approach in layered media was accomplished before by other researchers [23,24], simulation results suffer from several sources of numerical error due to aliasing, grid truncation, and nonlinearity parameters. While these problems are addressed in the research conducted by McGough et al. [14,25] for homogeneous media, in this study we developed a modified angular spectrum method that utilizes the approach to minimize numerical errors and extended it for nonhomogeneous layered media. The overall work of this study contains two novel aspects: (1) it proposes a method for focused ultrasound beam simulation in layered tissue media, and (2) it utilizes machine learning algorithms to model ultrasound responses. A similar research study was found in the literature where steady-state pressure and velocity field distributions in the thoracic aorta are predicted from existing computational data (CFD hemodynamic analysis of human blood vessels) using machine learning [26].

The machine learning approach presented in this study is expected to be useful when many quick simulations are required, and it will reduce numerical complexity and computational cost during ultrasound treatment planning.

## 2. Materials and Methods

### 2.1. Computation Approach

In the current study, a rectangular transducer surface is considered where ultrasonic array elements are arranged in the horizontal and vertical directions. The phase of these elements is determined using FOCUS software to obtain required distances [27]. The simulation is performed using an angular spectrum method that can address beam refraction and reflection effects in multiple tissue layers. Traditionally, the angular spectrum method is used only for homogeneous media, and for inhomogeneous tissue geometry, with different approaches found in the literature for realistic simulations [15,22,27]. Often, only homogeneous media are used to avoid complexities. To develop a machine learning model based on previous simulation data, we incorporated complexities and realistic scenarios (by adding tissue layers) even though simulation time is higher.

**Figure 1:**
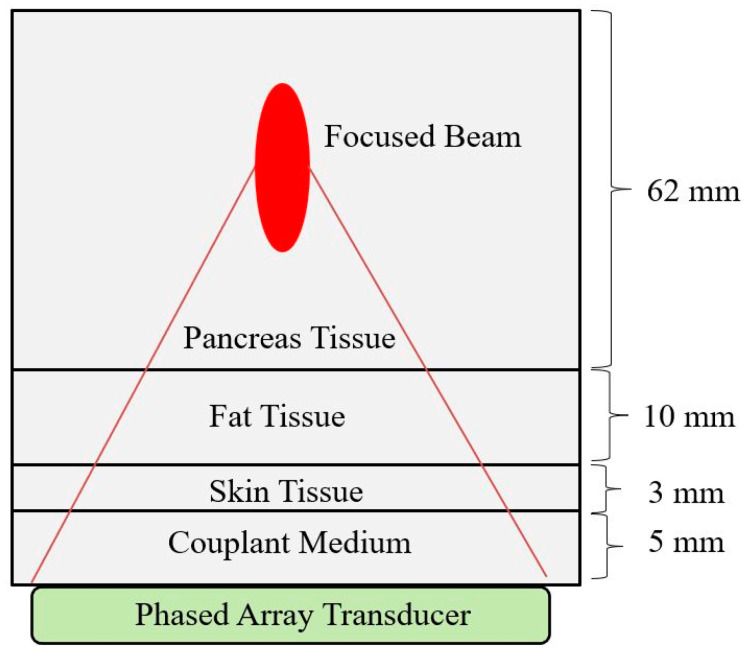
Focused ultrasound simulation through tissue layers consisting of couplant gel, skin, fat, and pancreas tissue. The simulation volume is 80 × 60 × 12 mm^3^.

The angular spectrum method developed by McGough et al. [14,25] was modified in this study so that it is suitable for layered media found in a typical tissue necrosis scenario. The simulation environment used for layered tissue media is shown in Figure 1, and the response found from the modified angular spectrum model is validated using a tissue necrosis experiment from the literature.

#### 2.1.1. Angular Spectrum Propagation Model

The angular spectrum is a widely accepted wave propagation model for focused ultrasound simulations [28,29]. It uses a source pressure plane, and from there, parallel layers of pressure planes are created using the 2D Fast Fourier Transform (FFT). We have used the Rayleigh–Sommerfeld integral to create the initial source pressure plane, and using this method a time-harmonic pressure field from a rectangular source is calculated by using Equation (1) [29].

(1) $$p(x,y,z;k)=\frac{j\rho cke^{j\omega t}}{2\pi }\iint_{s^{\prime }}v_{n}(r)\frac{e^{-jk|r-r^{\prime }|}}{\left|r-r^{\prime }\right|}ds$$

**Figure 2:**
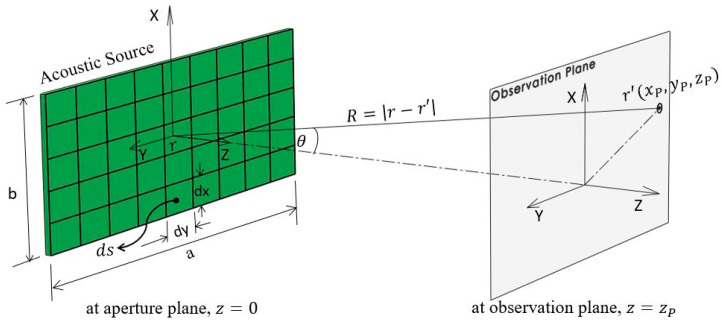
Rectangular transducer geometry and coordinate system for Rayleigh–Sommerfeld integral.

Here, $R=\left|r-r^{\prime }\right|=\sqrt{{\left(x_{P}-x_{0}\right)}^{2}+{\left(y_{P}-y_{0}\right)}^{2}+z_{P}{}^{2}}$ is the distance between the source point $r(x_{0},y_{0},0)$ and the observation point $r^{\prime }(x_{P},y_{P},z_{P})$, k is the wave number, *ω* is the excitation frequency, *ρ* is the medium density, and v is the normal particle velocity. The time-harmonic excitation is defined as $e^{j\omega t}$ where $j=\sqrt{-1}$, and t is the time. Figure 2 shows the rectangular transducer geometry and corresponding coordinate system.

The source pressure plane was generated at a quarter of the wavelength distance to avoid errors in the near-field, and from this source plane, parallel output pressure planes are calculated using Equation (2) [25,30].

(2) $$P\left(k_{x},k_{y},z\right)=P_{0}\left(k_{x},k_{y},z_{0}\right)H_{p}\left(k_{x},k_{y},\Delta z\right)$$

Here, $P_{0}\left(k_{x},k_{y},z_{0}\right)$ is the 2D Fourier transform of the input source pressure plane p(x,y,z;k) located at $z=z_{0}$, and $H_{p}\left(k_{x},k_{y},\Delta z\right)$ is a forward propagation transfer function in the spatial frequency domain as defined by Equation (3).

(3) $$H_{p}\left(k_{x},k_{y},\Delta z\right)=\{\begin{array}{c} e^{-j\Delta z\sqrt{k^{2}-k_{x}^{2}-k_{y}^{2}}}\;\;\;for\;k_{x}^{2}+k_{y}^{2}\leq k^{2}\\ e^{-\Delta z\sqrt{k_{x}^{2}+k_{y}^{2}-k^{2}}}\;\;\;for\;k_{x}^{2}+k_{y}^{2}>k^{2} \end{array}$$

To address the effect of reflections and transmission from different tissue boundaries, a 3D transmission coefficient matrix ($T_{P}$) is utilized, which implements the effect of acoustic impedances at each tissue volume.

(4) $$T_{p}=2\times {\left(\frac{\rho_{A}c_{A}\cos\theta_{out}}{\rho_{B}c_{B}\cos\theta_{in}}+1\right)}^{-1}$$

**Figure 3:**
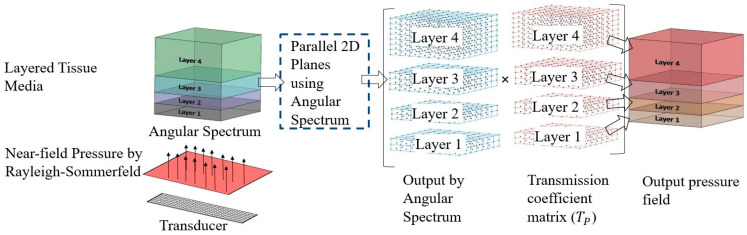
Schematic of 3D continuous-wave pressure field calculation in layered tissue media.

Here, $\theta_{in}$ and $\theta_{out}$ are incident and refraction angles, $\rho_{A}c_{A}$ and $\rho_{B}c_{B}$ are acoustic impedances in first and second media, respectively. The resultant pressure-field is calculated by multiplying angular spectrum pressure field with the 3D transmission coefficient matrix ($T_{P}$). After this operation, the updated simulation grid volumes for each tissue layer represent the resultant continuous-wave pressure field response, and a 2D slice (horizontal or vertical) of this field can be used to visualize the pressure field. Figure 3 represents a schematic of the 3D continuous-wave pressure field calculation procedure in layered tissue media using the modified angular spectrum method.

#### 2.1.2. Power Deposition and Temperature Rise

The pressure field obtained by the modified angular spectrum method can be used for finding power deposition and temperature fields. This requires further numerical processing of the pressure field. The acoustic intensity $I_{A}$ (W/m^2^) can be found from the resultant pressure field, p(x,y,x) using Equation (5). This intensity field is also known as the time-averaged rate of energy transmission, and it can be used to determine the power deposition field, Q using Equation (6) [31].

(5) $$I_{A}(x,y,z)=\frac{|p(x,y,z){|}^{2}}{2\rho_{0}c}$$

(6) $$Q\left(x,y,z\right)=2\alpha I_{A}\left(x,y,z\right)$$

Here $\rho_{0}$ is the density, c is the speed of sound in the tissue medium, and α is the attenuation coefficient.

The bio-heat transfer model is then used to find out the temperature field from the power deposition. Using this model, the temperature rise in the 3D simulation grid is calculated by using Equation (7) [32,33].

(7) $$\rho C\frac{\partial T}{\partial t}-k\nabla^{2}T=W_{b}C_{b}(T_{a}-T)+Q$$

Here, *C* is the tissue heat capacity, k is the tissue thermal conductivity, T is the time-dependent tissue temperature generated by power distribution Q, ρ is the density of the medium, $C_{b}$ is the specific heat of blood, $W_{b}$ is the blood perfusion rate, and $T_{a}$ is the arterial blood temperature, which is assumed to be 37 °C. The numerical approximation to find out steady-state temperature is determined by assuming boundary condition, ∂T/∂t = 0 [34,35,36].

#### 2.1.3. Model Validation

**Figure 4:**
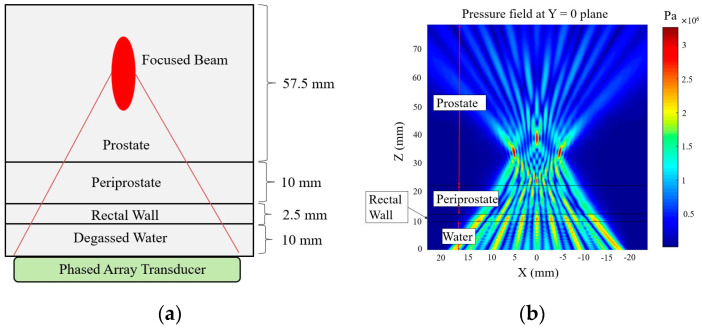
(**a**) Schematic of tissue layers for modified angular spectrum. (**b**) Pressure field simulation by focusing the beam at 40 mm depth, and 5, 0, and −5 mm azimuth inside prostate tissue with 990 × 1 phased array sonication.

An experimental tissue-heating scenario from the literature is used to validate the modified angular spectrum method. In this experiment, a commercial MR-guided endorectal ultrasound phased array transducer (ExAblate 2100, Insightec, LTD., Tirat Carmel, Israel) is evaluated through an ex vivo experiment [37]. The ultrasound phased array transducer used in the experiment, has 990 elements that are arranged linearly over 23 × 40 mm^2^ transducer area. The temperature rise inside the tissue volume is checked using an MR temperature monitoring system (3.0 T). We have used similar rectangular element sources, tissue layer thicknesses (Figure 4a), and properties (Table 1) to verify our model. The phased array excitation with an operating frequency of 2.3 MHz is used in the simulation to focus 40 mm deep inside the prostate tissue.

**Figure 5:**
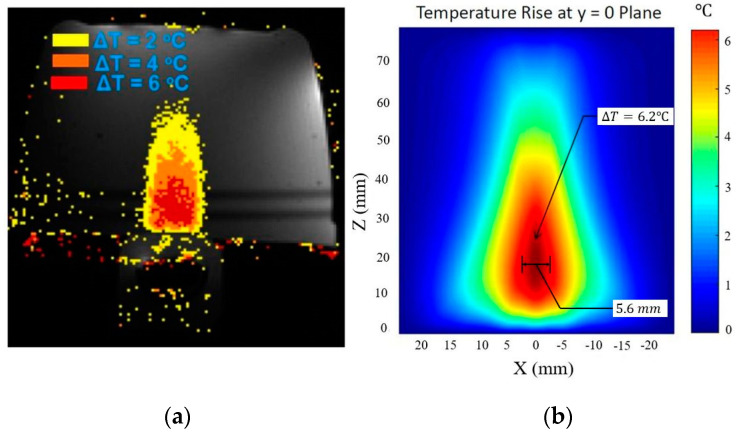
(**a**) Experimental observation of temperature rise in tissue-mimicking phantoms using Exablate 2100 [37]. (**b**) Temperature rise field by using the modified angular spectrum and bio-heat transfer model.

The schematic of the tissue layer setup is shown in Figure 4a. The pressure field inside the layered media produced by the modified angular spectrum continuous-wave sonication is shown in Figure 4b. The temperature field is calculated by using the bio-heat transfer model, and the maximum temperate rise found in the simulation is 6.20 °C, which was about 6 °C in the experiment. The width of 4 °C temperature rise contour is found to be 5.6 mm, which was recorded about 5 mm in the experiment. It is important to note that, in a previous study, we measured a similar temperature rise using the Rayleigh–Sommerfeld simulation and found close results (temperature rise 6.18 °C) [22]. These results are also consistent with another study where a finite element method is used to simulate the temperature rise of the current experimental scenario [38]. Figure 5 shows the experimental temperature rise using the MR thermometry and numerical simulation response. The tissue properties used in the simulation are given in Table 1.

### 2.2. Data Collection

A large volume of data is needed for training machine learning algorithms. Since it is not practical for us to use a very large number of transducers and produce focused ultrasound responses for different array distributions on the transducer surface, we generated data with the modified angular spectrum method that is developed and verified in this study (Section 2.1) as a substitution for real data. We have created a dataset by considering a common soft tissue necrosis scenario where the focused beam from a phased array transducer travels through layers of tissues before converging in the target media. The tissue layout shown in Figure 1 represents how the tissue layers are arranged through couplant media, skin, fat, and finally pancreas tissue. These tissue properties are given in Table A3.

A fixed rectangular transducer surface of 50 × 10 mm^2^ and a fixed kerf (space between elements) of 1 μm are maintained. The number of transducer elements is varied in horizontal and vertical directions, and for each of the transducer element combinations, the ultrasound beam is focused inside pancreas tissue from 25 to 75 mm. Thus, a rich dataset containing a total of 19,227 simulations is created. To get a relatively error-free result, we have used 3D calculation grids and approximately 20 to 30 min were spent for each of the simulations. The workstation specification for these simulations is given in the introduction section. Table 2 provides the range of parameters used for the focused ultrasound simulation dataset.

### 2.3. Data Preprocessing

The machine learning models are trained based on three inputs and three outputs parameters for each of the simulations. The input parameters are (1) number of ultrasonic elements in the X direction, (2) number of ultrasonic elements in the Y direction, and (3) focus distance. The output parameters are (1) maximum pressure, (2) maximum power, and (3) maximum temperature generated at the focus zone. To test the performance of trained algorithm, training and test data are separated (80% training and 20% testing). We have used “train_test_split” method from the “sklearn.model_selection” to split data arrays into random train and test subsets [43].

For the feature normalization, sklearn.preprocessing package from scikit-learn 0.24.1 is used, which rescales the range of data from minimum 0 to maximum 1 [44]. After that, the normalized data are fitted to transform training data for learning. This step is important so that the machine learning model is not biased towards particular features of the dataset. 

### 2.4. Machine Learning Models

Recently, advanced machine learning models have been widely analyzed and have found success in many non-conventional prediction models [45,46,47]. In this study, several machine learning models are considered and analyzed for focused ultrasound data set. Before testing out these models, we evaluated the performance of an ordinary multivariable linear regression model. In this approach, the training X and Y element data are used to determine coefficients $w=\left(w_{1},w_{2},w_{3},\ldots \ldots \ldots .,w_{n}\right)$. The w’s need to be chosen by this algorithm to minimize the residual sum of squares between that targets and predicted values. If $\hat{y}$ is the predicted value for a particular X, Y element, and focus distance; it can be defined by using Equation (8).

(8) $$\hat{y}\left(w,x\right)=w_{0}+w_{1}x_{1}+\ldots \ldots \ldots +w_{n}x_{n}$$

Here, w act as coefficient vector and $w_{0}$ act as intercept. The loss function, f that needs to be minimized is given by Equation (9) [48]:

(9) $$f=\overset{n}{\underset{i}{\sum }}{\left[error\right]}^{2}=\overset{n}{\underset{i}{\sum }}{\left(y_{i}-{\hat{y}}_{i}\right)}^{2}$$

To capture the nonlinear relationships between features and outcomes, several machine learning models are tested besides the statistical regression model. The grid search optimization algorithm was used with 5-fold cross-validation to choose the adjustable parameters of the machine learning models. We have used “GridSearchCV” class from “sklearn.model_selection” to find these parameters [49]. The cross-validation error and accuracy in grid-search are given in Table A1 (Appendix A). A brief description of each of the machine learning models used and the corresponding implementation methods are discussed in the following subsections.

#### 2.4.1. Decision Tree Algorithm

**Figure A1:**
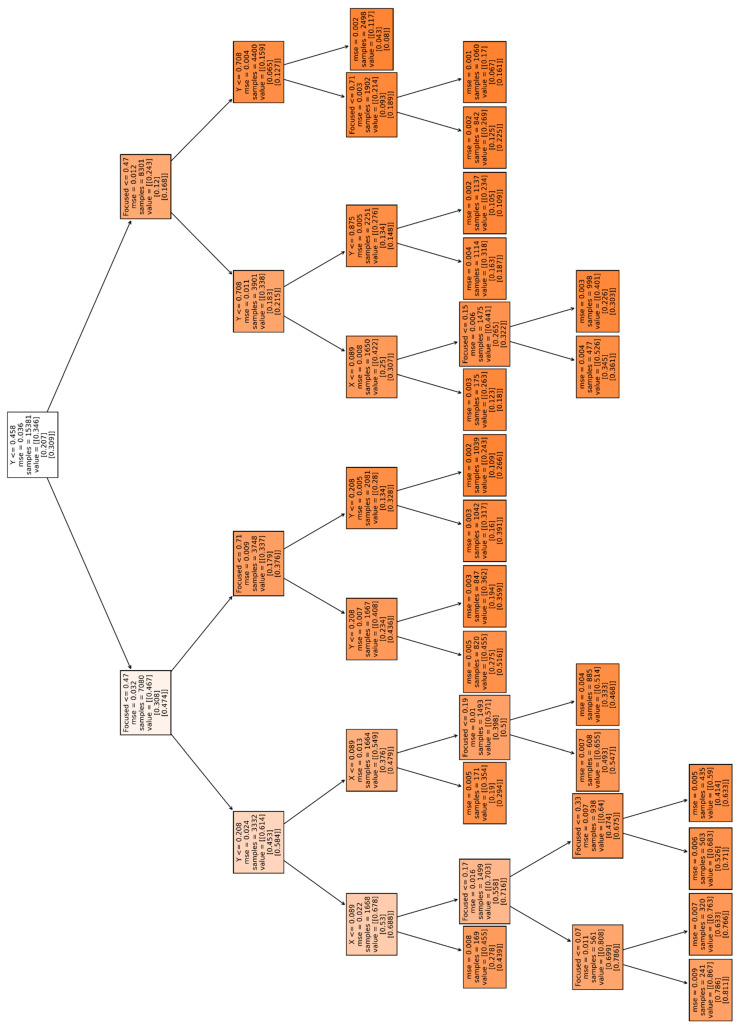
Decision tree for predicting maximum pressure, power, and temperature at the focused zone.

The decision tree algorithm maps a reasoning process resembling a tree-like structure and is generally suitable for multi-output problems. We have used a non-parametric supervised decision tree algorithm, Classification and Regression Tree (CART), to predict maximum pressure, power, and temperature at the focused zone. The CART algorithm partitions the feature space into local regions via a sequence of recursive split and then fit model (piecewise constant approximation) in each one [50]. The goal of using this algorithm is to minimize the RSS (Residual Sum of Square) errors between the observed and the mean in each node, such as the loss function f in Equation (9). The number of leaf nodes that give optimum output is 20 in the training process. The decision tree of the CART algorithm in our dataset is given in Figure A1 (Appendix A).

#### 2.4.2. Support Vector Regression (SVR)

The Support Vector Regression (SVR) algorithm is another technique for regression problems. The SVR tries to find a function $\hat{y}=f\left(x\right)$ that has at most ε (a prescribed parameter by algorithm) deviation from the actually obtained targets y for all the training data. In this algorithm, any deviation less than ε is ignored, and any deviation larger than ε will be treated as a regression error. Therefore, a trade-off between the fitting accuracy and prediction accuracy is implemented, and the threshold is used to zero out training data fitting errors [51]. The loss function used in SVR can be described using Equation (10) [52].

(10) $${\left|y-f\left(x\right)\right|}_{\varepsilon }≜\{\begin{array}{cc} 0 & if\;\left|y-f\left(x\right)\right|\leq \varepsilon \\ \left|y-f\left(x\right)\right|-\varepsilon & otherwise \end{array}$$

Here, the loss function defines an ε−insensitivity zone (also known as ε-tube), and an increase in ε (reduction in accuracy requirements) results in smoothing effects on modeling noisy data.

SVR is more robust than other algorithms that use Sum of Squared Error (SSE) based criteria and is also more tolerant to noises in the dataset. It has been successfully trained on many different types of data sets. 

To assess the applicability of SVR on the focused ultrasound dataset, we have evaluated it for developing the response model. The LIBSVM library package of scikit-learn 0.24.1 is used to implement this algorithm [43]. In addition, the model is trained separately for each of the outputs (maximum pressure, maximum intensity, and maximum temperature) for corresponding inputs. After training for prediction, target columns (3 outputs) are stacked to obtain the final set of outputs.

The adjustable parameters of SVR model were selected using the grid-search algorithm so that the parameters are optimized by cross-validation [53]. For the maximum pressure and maximum temperature prediction, the Radial Basis Function (RBF) kernel was used with the regularization parameter, C=5, epsilon = 0.1 (epsilon-tube within which no penalty is associated in the training loss function), and kernel coefficient gamma = 1/(num of features * X.var()) [43]. For the maximum power prediction, the radial basis function (RBF) kernel was used with the regularization parameter *C* = 100, epsilon = 0.1, and gamma = 1/(num of features).

#### 2.4.3. Random Forest Regression 

Random forest is an ensemble machine learning method in which a model makes more reliable decisions to create a combination of outputs of many different decision trees. Overall, it builds upon a forest of random decision trees, where each tree grows separately [54]. Here, many decision trees are constructed (in a forest) and parallelly trained to act as a group of learners. After that, a classifier, also known as the random classifier, is used to vote and determine the final class of the tree. For this unique method, random forest is suitable for very large datasets and typically does not overfit [55,56]. Since this algorithm uses many decision trees simultaneously, more computation power is required, but on the other hand, it gives higher accuracy. In our analysis, we have used “sklearn.ensemble method” from scikit-learn 0.24.2 to implement the random forest algorithm. The optimum number of trees in the forest (estimator) is found to be 1000. To control both the randomness and bootstrapping of the samples when building trees, random state method is used [57], and the sampling of the features were considered for the best split at each node.

### 2.5. Performance Metrics

To evaluate the performance of the model, Root Mean Squared Error (RMSE), R-Squared (R^2^), Akaike Information Criterion (AIC), and Bayesian Information Criterion (BIC) parameters are analyzed. The RMSE is the square root of the Mean Squared Error (MSE) and can be determined using Equations (11) and (12).

(11) $$MSE=\frac{1}{n}\overset{n}{\underset{i}{\sum }}{\left(y_{i}-{\hat{y}}_{i}\right)}^{2}$$

(12) $$RMSE=\sqrt{MSE}$$

To identify the proportion of variation in the outcome, R-Squared (R^2^) or the coefficient of determination is calculated, which is also an indication of goodness of fit and gives an insight into how well unknown samples are likely to be predicted. For $y_{i}$ and ${\hat{y}}_{i}$ being the true value and predicted value of i-th data point respectively, the estimated R^2^ is defined using Equation (13) [58].

(13) $$R^{2}\left(y,\hat{y}\right)=1-\frac{\sum_{i=1}^{n}{\left(y_{i}-{\hat{y}}_{i}\right)}^{2}}{\sum_{i=1}^{n}{\left(y_{i}-\bar{y}\right)}^{2}}$$

where, $\bar{y}=\frac{1}{n}\sum_{i}^{n}y_{i}$.

Other relevant metrics, Akaike Information Criterion (AIC) and BIC (Bayesian Information Criterion) are used in our evaluation. AIC and BIC are considered as unbiased estimates of the model’s error prediction. They use the model’s maximum likelihood estimation using the following Equations (14) and (15) [59].

(14) AIC=−2ln(L)+2k

(15) BIC=−2ln(L)+2ln(N)k

where, *L* = value of likelihood and *k* = number of estimated parameters.

*AIC* is low for models with high log-likelihoods but adds a penalty parameter for models with more parameters that likely to overfit. In general, a lower *AIC* and *BIC* indicate a better fit. The optimal model should be chosen based on the highest R^2^ and minimum *AIC* and *BIC* values.

Other performance metrics that give unbiased estimates, such as AICc (*AIC* corrected) and Mallows Cp, are directly related to the *AIC* values. Therefore, AICc and Mallows Cp metrics are not considered in our study.

## 3. Results

### 3.1. Inference on Test Data

The performance of the decision tree (CART), SVR, and random forest in predicting maximum pressure, intensity, and temperatures are given in Table 3. A result from ordinary multivariable regression is included in this table for making comparisons. In our analysis, the random forest gives superior statistical performance with an R^2^ value of 0.9997, RMSE of 0.0032, AIC of −44164.63, and BIC of −44145.87. Since it will be unreasonable to find a better result than the random forest, other advanced machine learning models such as xgBoost and LightGBM are not considered in our study.

The results presented in Table 3 indicate that the random forest algorithm is most suitable for the focused ultrasound dataset, and we can use this model as a prediction tool for maximum pressure, intensity, and temperatures during ultrasound therapy. All evaluation metrics in Table 3 are evaluated based on the testing dataset, which is 20% of the total number of simulations. The AIC and BIC metrics are used to evaluate the relative quality of the models. Using the random forest model, we achieved the highest R^2^, and lowest AIC and BIC values, which is also a strong indication of a better fit model.

### 3.2. Inference on External Data

To examine the model’s performance on unknown data, which is not part of the focused ultrasound dataset, we have used 10 simulations using random X, Y elements and focus distances (Table A2). For these simulations, the data points we have in the training and testing phase were avoided. The performance of the evaluated models on these external data is shown in Table 4.

For these external data, the best performance is found for random forest algorithm with an R^2^ value of 0.9970, RMSE of 0.0123, AIC of −82.56, and BIC of −81.65. It is noted that in external data, multiple linear regression is performing better than the decision tree algorithm due to the small volume of data. Table A2 in Appendix A compares the maximum pressure, power, and temperatures in the external data points by modified angular spectrum simulation and the random forest prediction model.

## 4. Discussion

Predicting maximum pressure, power deposition, and temperature rise in the focus during ultrasonic surgery or hyperthermia is a very critical part of treatment planning. Numerical simulation is one of the best approaches for making such predictions. Due to the complexity of the simulation environment, many different approximations of numerical models are utilized, but the overall simulation time remains relatively high. Often, therapeutic simulations are conducted on a similar patient model. Therefore, a fast simulation method based on existing reference data would facilitate the process of determining the responses during medical treatment planning. A fast prediction model can also help surgeons to make a quick decision by comparing different treatment options.

In this study, we presented an alternative approach for modeling these responses using machine learning algorithms. The dataset on which machine learning algorithms are trained is created using a modified angular spectrum method developed in this study, and the validity of the simulation approach is checked by using an experimental study found in the literature. All the simulations were performed by focusing ultrasound beam inside pancreas tissues.

The focused ultrasound dataset contains simulations for different X elements, Y elements, and focus distances. Several predictive models (both statistical and machine learning) are evaluated, and the machine learning algorithm, random forest was found to be the best one in our study. To further check the model performance, we used external random simulations that were not part of the focused ultrasound dataset but within the ranges of the original dataset: X elements ranging from 16 to 128, Y elements ranging from 16 to 64, and focus distances from 25 to 75 mm. In the external dataset, the random forest algorithm demonstrated superior performance compared to other models, and Table A2 (Appendix A) shows a comparison of the random forest model with numerical simulation results. 

In previous studies, we demonstrated that if we change the simulation tissue properties, the maximum pressure, power, and temperatures field patterns shift up or down in values but follow a similar pattern in a 3D space [22,60]. Therefore, it is reasonable to conclude that if we enrich the dataset by adding different tissue properties and transducer geometries, the random forest model will remain very effective in modeling focused ultrasound responses.

## 5. Conclusions and Future Work

The current study presents a model that is trained on a very large dataset (19,227 focused ultrasound simulations). The efficacy of the model is tested using standard statistical performance metrics, such as RMSE, R^2^, AIC, and BIC. It is noted that a machine learning model does not perform well outside of the range of the dataset. So, an extension of this dataset covering most of the commercial transducer surface area and element distributions found in the soft tissue necrosis scenario will be required for more robust model performance.

In the future, we plan to extend this dataset by incorporating wide-ranging transducer surface, element distributions, tissue layer thicknesses, and properties. With further data inclusion, the applicability of this method can become more valuable in practical ultrasound treatment planning.

## Figures and Tables

**Table 1:** Tissue properties used for degassed water, rectal wall, preiprostate, and prostate media [39,40,41,42].

Parameters	Unit ^a^	Coupling Medium (Degassed Water)	Rectal Wall	Periprostate	Prostate
Sp. Heat capacity of blood	J/kg-K	3480	3720	3720	3720
Blood perfusion	Kg/m^3^-s	0	4	5	2.5
Density	Kg/m^3^	1000	1060	1060	1060
Speed of sound	m/s	1480	1500	1500	1500
Power law exponent	unitless	2	1	1	1
Attenuation	dB/cm-MHz	0.00025	0.5211	0.4343	0.504
Sp. Heat of medium	J/kg-K	4180	3500	3500	3600
Thermal conductivity	W/m-K	0.615	0.56	0.50	0.50
Nonlinearity parameter	unitless	0	1	1	1

^a^ Units are the same as International System Units (SI); J = Joule, kg = kilogram, K = kelvin, m = meter, s = second, dB = decibel, cm = centimeter, MHz = megahertz, W = watt.

**Table 2:** Range of ultrasonic transducer elements and focus distances for constant transducer area and kerf.

X Elements	Increment Along X	Y Elements	Increment Along Y	^1^ Focus Distance (mm)	Focus Increment (mm)
16 to 128	4	16 to 64	4	25 to 75	1

^1^ Ultrasound beam is focused inside the target (pancreas) tissue from the transducer surface.

**Table 3:** Performance comparison of machine learning models for test data.

Model	RMSE	R^2^	AIC	BIC
Multiple Linear Regression	0.0708	0.8554	−20,410.13	−20,391.36
Decision Tree	0.0587	0.9045	−21,858.23	−21,839.46
Support Vector Regression	0.0484	0.9330	−23,301.89	−23,283.13
Random Forest	0.0032	0.9997	−44,164.63	−44,145.87

RMSE = Root Mean Squared Error, R^2^ = R-Squared, AIC = Akaike Information Criterion, and BIC = Bayesian Information Criterion.

**Table 4:** Performance comparison of machine learning models for external data.

Model	RMSE	R^2^	AIC	BIC
Multiple Linear Regression	0.0548	0.9412	−52.74	−51.84
Decision Tree	0.0641	0.9195	−49.29	−48.38
Support Vector Regression	0.0363	0.9707	−61.69	−60.79
Random Forest	0.0123	0.9970	−82.56	−81.65

RMSE = Root Mean Squared Error, R^2^ = R-Squared, AIC = Akaike Information Criterion, and BIC = Bayesian Information Criterion.

## Data Availability

Data is available from the corresponding author upon reasonable request.

## Appendix A

**Table A1:** The cross-validation error and accuracy in grid-search.

	SVR			Decision Tree	Random Forest
	Pressure	Power	Temperature Rise		
RMSE	0.0448	0.0507	0.0491	0.0588	0.0042
R^2^	0.9455	0.9113	0.9426	0.9038	0.9995

RMSE = Root Mean Squared Error, R^2^ = R-Squared.

**Table A2:** Comparison of random forest model with angular spectrum method at 10 random and external data points.

				Angular Spectrum			Random Forest		
	No. of X Elements	No. of Y Elements	Focus Depth	Maximum Pressure	Power Deposition	Temp. Rise	Maximum Pressure	Power Deposition	Temp. Rise
^a^ Units			mm	MPa	KW/m^2^	°C	MPa	KW/m^2^	°C
	68	21	50.00	4.862	707.630	45.516	4.913	722.746	46.645
	31	63	57.00	2.008	120.799	9.212	1.969	116.171	8.812
	37	40	68.40	2.677	214.536	19.918	2.701	218.420	20.075
	57	21	46.28	5.298	840.119	50.037	5.398	872.386	51.704
	72	17	46.98	5.383	867.439	52.269	5.438	885.294	53.389
	46	27	35.80	5.673	963.298	48.749	5.543	919.806	46.990
	32	46	68.33	2.413	174.412	16.103	2.510	188.719	17.332
	62	49	36.19	4.004	479.941	24.126	4.074	496.900	24.944
	19	55	61.63	1.977	117.040	10.372	1.969	116.239	10.168
	33	21	29.45	6.1330	1125.80	51.030	6.147	1133.200	51.471

^a^ Units: mm = millimeter, MPa = mega pascal, KW = kilo watt, °C = degree centigrade.

**Table A3:** Selected properties of tissues for focused ultrasound simulation in layered media [61,62,63,64,65,66,67].

Parameters	Unit (SI) ^a^	Coupling Medium	Skin	Fat	Pancreas
Sp Heat capacity of blood	J/kg-K	3480	3480	3480	3480
Blood perfusion	Kg/m^3^-s	0	5	0.54	10
Density	Kg/m^3^	1033	1200	950	1050
Speed of sound	m/s	1490	1560	1478	1591
Power law exponent	unitless	2	2	1.4	0.78
Attenuation	dB/cm-MHz	0.58	2.5	0.61	0.955
Sp Heat of medium	J/kg-K	3960	3400	3800	3160
Thermal conductivity	W/m-K	0.5574	0.23	0.217	0.547
Nonlinearity parameter	unitless	0.35	4.435	5.5	2.85

^a^ Units are the same as International System Units (SI); J = Joule, kg = kilogram, K = kelvin, m = meter, s = second, dB = decibel, cm = centimeter, MHz = megahertz, W = watt.
